# From Microbiota Correction to Host Protection: A New Therapeutic Target for the Prevention and Treatment of Postoperative Complications

**DOI:** 10.3390/jcm15135161

**Published:** 2026-07-02

**Authors:** Zelimkhan Berikkhanov, Miroslava Pilipenko, Elizaveta Ermakova, Maria Sukhanova, Milena Ivanova, Aleksey Kotelnikov, Andrey Nikolaev, Vadim Razumovsky, Vladislav Rakintsev, Alexey Shestakov, Evgeniy Tarabrin, Sergey Muraviev

**Affiliations:** 1 Department of Hospital Surgery No. 2, Sechenov University, 119991 Moscow, Russia; berikkhanov_z_g@staff.sechenov.ru (Z.B.); ermakova.e.d@mail.ru (E.E.); sukhanova_m_a@student.sechenov.ru (M.S.); ivanova_m_yu_1@staff.sechenov.ru (M.I.); kotelnikovag@mail.ru (A.K.); nikolaev_a_m@staff.sechenov.ru (A.N.); razumovskiy_v_s@staff.sechenov.ru (V.R.); shestakov_a_l@staff.sechenov.ru (A.S.); tarabrin_e_a@staff.sechenov.ru (E.T.); muravev_s_yu@staff.sechenov.ru (S.M.); 2 Surgical Department No. 3, Sechenov University, 119991 Moscow, Russia; rakintsev_v_s@staff.sechenov.ru

**Keywords:** gut microbiota, dysbiosis, postoperative complications, intestinal barrier, surgical stress, bacterial translocation, intestinal epithelium, narrative review

## Abstract

**Background/Objectives.** The intestinal microbiota is a key contributor to postoperative complications, yet direct interventions targeting dysbiosis—antibiotics, probiotics, and synbiotics—have produced inconsistent results. This paradox indicates a fundamental gap in understanding host–microbiota interactions under surgical stress. We aimed to re-examine the causal role of dysbiosis in postoperative pathogenesis and propose a revised therapeutic paradigm centered on host barrier protection. **Methods.** A narrative literature review was conducted, searching PubMed/MEDLINE, Scopus, and Web of Science for articles published between 2009 and 2025. Reference lists of included publications were additionally screened. Studies in English and Russian were eligible; 107 references were included. **Results.** We hypothesize that dysbiosis in surgical patients may, at least in part, represent a predictable ecological response to systemic hypoperfusion, pharmacological burden, and ischemia–reperfusion injury, rather than acting solely as an independent pathogenic agent. Microbial shifts, characterized by the depletion of short-chain fatty acid-producing commensals and the expansion of pathobionts, frequently accompany epithelial injury; however, available human data are predominantly observational and do not permit definitive determination of the temporal sequence. This hypothesis provides the conceptual foundation for the proposed therapeutic reorientation. **Conclusions.** The present findings support the rationale for transitioning from microbiome manipulation to a “host-first” strategy, which prioritizes the restoration of intestinal barrier integrity through the administration of cytoprotective agents and targeted metabolic substrates (glutamine and butyrate). We propose the Gut Resilience Index (GRI) as a theoretical construct to identify patients approaching a critical threshold necessitating rescue therapy. It must be emphasized that both the “host-first” strategy and the GRI remain hypothetical frameworks requiring prospective validation. The most critical next steps include the development and validation of the GRI in prospective cohort studies, as well as randomized controlled trials directly comparing barrier-oriented strategies with standard care.

## 1. Introduction

Postoperative complications such as anastomotic leakage, intestinal obstruction, surgical site infections, and sepsis remain among the most serious challenges in modern surgery. In recent years, an increasing number of studies have confirmed that the intestinal microbiota plays a key role in these processes [1]. It has been demonstrated that systemic and intestinal stress resulting from surgical intervention and perioperative therapy is highly likely to induce dysbiosis. The main features of dysbiosis include reduced microbial diversity, a decrease in beneficial commensal populations, and an excessive growth of opportunistic pathogens [2,3,4]. These characteristics are strongly associated with the risk of developing severe complications.

This association has led to numerous attempts to directly correct dysbiosis using antibiotic therapy or, more recently, with what is considered a more promising approach: probiotics, prebiotics, and synbiotics [5,6]. However, the clinical results of these interventions have been highly inconsistent. Antibiotic therapy frequently leads to complications such as antibiotic-associated diarrhea, antibiotic resistance, and secondary infections. Probiotics, in turn, have shown beneficial effects in some studies while demonstrating no impact or even adverse outcomes in others [5]. This raises a fundamental question: if we can so clearly describe the composition of a “pathological” microbiota, why do our attempts to modulate it under conditions of acute surgical stress so often fail?

We hypothesize that this therapeutic paradox stems from a misformulated problem. The issue is not that pathogenic bacteria attack the host and directly cause dysfunction. Rather, we hypothesize that dysbiosis is a consequence, not a cause, of systemic stress—specifically, global hypoperfusion and inflammation—that render the intestinal environment hostile to symbiotic commensals. In this conceptual model, the microbiota might be acting not as a primary aggressor but as a sensitive marker, a “mirror” of the host’s physiological state and, subsequently, as a potent mediator that exacerbates the initial injury. As a mediator, the microbiota becomes an independent pathological focus whose effects add to the systemic stress, thereby worsening the clinical course. In this context, inappropriate treatment can amplify stress exponentially, potentially leading to critical or even fatal outcomes.

The objective of the present work is not to review taxonomic shifts, but rather to systematically reevaluate the existing data to develop a novel conceptual model of host–microbiota interactions under stress conditions. To this end, we propose the following: (1) to investigate how systemic factors predictably alter the microbial community and to evaluate the evidence supporting its role as a biomarker; (2) to analyze the mechanisms by which the altered microbiota may exacerbate the pathological process, potentially acting as a mediator within a “vicious cycle” of complications; and (3) based on this analysis, to propose a fundamental shift in the therapeutic paradigm—from direct manipulation of the microbiota to a strategy focused on the protection and restoration of the intestinal barrier, thereby fostering conditions for the natural recovery of the entire ecosystem. We emphasize that the proposed causal model is advanced as a testable hypothesis, rather than an established pathophysiological doctrine.

## 2. Materials and Methods

A narrative literature review was conducted to critically analyze current evidence on the role of the gut microbiota and intestinal barrier dysfunction in the development of postoperative complications. The following databases were searched: PubMed/MEDLINE, Scopus (Elsevier), Web of Science (Clarivate), eLIBRARY.RU, and CyberLeninka. Search terms were used in Boolean combinations (AND/OR) and included both MeSH headings and free-text queries: “gut microbiota,” “intestinal microbiome,” “dysbiosis,” “postoperative complications,” “surgical stress,” “intestinal barrier,” “epithelial barrier function,” “tight junction,” “bacterial translocation,” “enteral insufficiency,” “anastomotic leakage,” “surgical site infection,” “sepsis,” “probiotics,” “prebiotics,” “synbiotics,” “short-chain fatty acids,” “butyrate,” “Faecalibacterium prausnitzii,” “Akkermansia muciniphila,” “Fusobacterium nucleatum,” “Enterococcus,” “colorectal surgery,” “cardiac surgery,” “gastrointestinal surgery,” “ischemia-reperfusion,” “gut–heart axis,” “host–microbiota interaction,” and “intestinal resilience.”

Publications from January 2009 to October 2025 were considered; seminal papers published before 2009 were included when cited by multiple contemporary sources and deemed conceptually significant to the field. Both English- and Russian-language articles were eligible; Russian-language publications (refs. 2;7;8;9;34;47;50;51;53;99;100;101) were included to incorporate regional clinical data and guidelines unavailable in international databases.

Eligible study types included original research articles, narrative reviews, systematic reviews, meta-analyses, and clinical guidelines addressing gut microbiota composition in the context of surgical procedures, perioperative care, or postoperative complications, as well as studies on intestinal barrier function, bacterial translocation, and host–microbiota interactions under stress conditions. In vitro, animal, and human studies were all considered, given the conceptual nature of this review. Conference abstracts without full-text publication, studies focused exclusively on pediatric populations, and studies unrelated to the surgical or acute stress context were excluded.

Titles and abstracts were screened independently by two authors; full texts of potentially relevant records were then reviewed. Disagreements regarding inclusion criteria or data interpretation were resolved by discussion and consensus; when consensus could not be reached, the senior author made the final decision.

As this is a narrative review, no formal risk of bias assessment or quantitative quality appraisal was performed. Nevertheless, study design, methodological rigor, sample characteristics, reproducibility of findings, and consistency with the existing literature were considered during data interpretation and synthesis.

When multiple publications addressed the same patient population, overlapping datasets, or similar research questions, preference was given to the most comprehensive, current, and methodologically sound reports to avoid double-counting of data.

Reference lists of included articles were manually searched for additional relevant sources. A total of 107 references were included. No PRISMA flow diagram was produced, and no review protocol was registered, as this review was designed to support a conceptual reappraisal of the existing evidence base rather than to quantify aggregate effect sizes.

To avoid ambiguity, we provide working definitions of the key conceptual terms used throughout this review. These terms reflect the authors’ own conceptual framework, developed through clinical and scientific collaboration and endorsed by external experts who agreed with our interpretation. We recognize that our definitions may be contested by the wider scientific community and make no claim to finality; we welcome public discussions and critical responses, as both would contribute to a more precise characterization of the concepts we introduce.

*The “host-first” strategy* refers to a therapeutic approach that prioritizes protection and restoration of the structural and functional integrity of the intestinal wall—including microcirculation, enterocytes, and intercellular junctions—over direct correction of microbiota composition. Within this framework, the microbiota is viewed as a dependent variable responding to changes in its habitat, not as an independent therapeutic target. The principal instruments include cytoprotective agents, metabolic substrates (glutamine and butyrate), and optimization of hemodynamics and blood rheology.

*Gut resilience* denotes the capacity of the intestinal wall and its microbial ecosystem to maintain or restore homeostasis, barrier function, and a normal microbial community following exposure to a stressor such as surgical trauma, ischemia, inflammation, or pharmacological intervention. Resilience is determined not by individual components but by the integrative properties of the system as a whole: adequate microcirculation, intact intercellular junctions, functional enterocyte activity, and ecological stability of the microbial community.

*The tipping point (point of no return)* is a hypothetical threshold of intestinal ecosystem degradation beyond which endogenous recovery becomes impossible, and the intestine transitions from a passive target into an active “engine” of multiple organ failure. The existence of this threshold is currently a working hypothesis requiring empirical verification.

*Rescue therapy* refers to aggressive interventions applied in patients presumed to have crossed the tipping point—including targeted antibiotics, fecal microbiota transplantation (FMT), or other methods of direct microbial community correction. The goal is not restoration of the original equilibrium but prevention of catastrophic collapse of host physiology. This concept is hypothetical, and lacks validated clinical protocols.

*Ecosystem collapse* describes a state of irreversible destabilization of the intestinal microbial community, characterized by a sharp decline in alpha diversity, disappearance of dominant commensals (particularly butyrate-producing anaerobes), and expansion of pathobionts. In the context of this review, ecosystem collapse is viewed not as a primary event but as a consequence of profound intestinal wall injury that renders the habitat inhospitable to symbiotic microbiota.

## 3. Results

### 3.1. Changes in the Microbiota Under Adverse Conditions

Let us examine how systemic stress affects the composition of the intestinal microbiome. Systemic triggers in this context include surgical intervention, perioperative pharmacotherapy (including antibiotic administration and other drugs), mechanical bowel preparation, postoperative fasting, and certain pathological conditions of the gastrointestinal tract.

Before proceeding to the analysis of these associations, a critical caveat is warranted. The vast majority of studies in humans documenting microbial shifts in surgical patients employ a cross-sectional or observational design. While these studies can identify correlations between microbiota composition and clinical outcomes, they cannot definitively establish whether dysbiosis precedes, follows, or occurs concurrently with intestinal wall injury. The temporal sequence—and thus the direction of causality—remains inferential in most clinical contexts, except for the specific obstructive and ischemic models discussed below. Throughout this review, when we characterize dysbiosis as a “consequence” of systemic stress, we do so as a working hypothesis rather than an established fact.

The term “surgical intervention” encompasses not only gastrointestinal procedures. Our analysis identified studies demonstrating a clear association between dysbiosis and cardiac surgery. Open-heart operations, particularly those performed with cardiopulmonary bypass, elicit a strong systemic inflammatory response and intestinal hypoperfusion (ischemia–reperfusion injury) [7,8,9,10]. Although the surgical field is anatomically distant from the abdominal cavity, these procedures lead to dysbiotic alterations characterized by a reduction in protective commensal populations and an overgrowth of opportunistic microorganisms (pathobionts). Moreover, the degree of dysbiosis directly correlates with the risk of postoperative infections and multiple organ failure, vividly illustrating the close connection along the “gut–heart axis” and reinforcing the concept of the microbiota as a reflection of the host’s systemic condition [8,9].

The most informative data on the temporal relationship between intestinal wall injury and microbial alterations have been derived from two classic clinical models: acute bowel obstruction and mesenteric thrombosis. In acute bowel obstruction, the initiating event is a mechanical obstruction that increases intraluminal pressure and directly impairs submucosal microcirculation. Within the first 2–4 h, mucosal ischemia with enterocyte hypoxia develops; over the subsequent 4–6 h, progressive disruption of intercellular contacts and enterocyte desquamation occur, leading to the formation of basement membrane defects. Only after these structural injuries are established can luminal microorganisms penetrate the defect via a pathological paracellular pathway—a mechanism fundamentally distinct from the physiological transcellular transport across M cells in the intact intestine. Experimental evidence indicates that antibiotic prophylaxis initiated within the first 2 h can delay, but not prevent, bacterial translocation after 6 h of ischemia. This observation is consistent with the interpretation that structural bowel wall injury represents the primary event in the pathophysiological cascade, whereas microbial invasion occurs secondarily. A similar sequence is observed in mesenteric thrombosis: vessel occlusion → wall ischemia → enterocyte necrosis → secondary microbial colonization of necrotic areas. Early revascularization within the “golden 6 h”—even in the presence of an already altered microbiota—preserves bowel viability and minimizes complications, whereas intervention after the development of transmural necrosis renders any microbiome-directed therapy futile. These models provide a plausible pathophysiological substrate for a causal hierarchy in which host injury precedes secondary microbial shifts. However, it is crucial to emphasize that these conclusions are derived from highly specific and extreme clinical scenarios. For most other surgical contexts—such as elective resections, anastomosis formation, and cardiac surgery—the detailed temporal profiles of the “intestinal wall–microbiota” interaction remain poorly understood. Whether this same sequence applies universally, or whether reciprocal or simultaneous mechanisms operate in less difficult situations, remains an open question requiring further investigation using advanced in vivo imaging techniques and serial biopsies.

Turning to the specific alterations of the intestinal microbiota, the most sensitive microbial groups, those dependent on stable pH and oxygen gradients, are affected first. There is a systemic decline in the abundance of obligate anaerobes producing short-chain fatty acids (SCFAs), particularly butyrate, which serves as the primary energy source for colonocytes and a key regulator of anti-inflammatory protein expression [2,3,11]. Populations of *Faecalibacterium prausnitzii*, *Roseburia*, and clusters *Clostridium* IV and XIVa (including *C. coccoides* and *C. leptum*) are markedly reduced, directly weakening the metabolic defense of the intestinal barrier [1,12,13,14,15,16,17,18,19,20,21,22,23]. The abundance of the families *Lachnospiraceae* and *Ruminococcaceae* also decreases [1,15,20,24,25,26]. Following surgical intervention, perioperative therapy, ischemia–reperfusion injury, or inflammatory bowel disease (IBD), the total counts of *Bifidobacterium*, *Peptostreptococcus*, *Lactobacillus*, and *Bacteroides* may significantly decline [14,16,17,18,19,22,25,26,27,28,29,30,31,32,33,34,35].

The ecological niches vacated by these protective species are rapidly occupied by more resilient pathobionts. Aging and surgical stress are both associated with an increased proportion of *Proteobacteria*, members of which secrete lipopolysaccharides (LPSs) that promote inflammation [36,37]. There is an expansion of *Enterobacteriaceae,* particularly *Escherichia coli* and *Klebsiella* spp., as well as *Enterococcus* and *Staphylococcus*, all of which are linked to surgical site infections (SSIs) and anastomotic leakage [1,4,15,19,22,24,25,26,27,29,34,35,36,38,39,40,41,42]. The abundance of *Fusobacterium nucleatum,* an oral commensal acting as an oncogenic pathogen when translocated to the gut, increases as well [2,18,19,29,31]. Additionally, elevated levels of *Acinetobacter lwoffii*, *Hafnia alvei*, and enterotoxigenic *Bacteroides fragilis* (ETBF) have been reported, all associated with postoperative complications and chronic inflammation [18,19,20,43]. The key microorganisms discussed in this section are grouped by functional role and clinical significance in Table 1.

Thus, the observed microbial shifts appear to represent not a chaotic process, but rather an orderly and predictable ecological response to deteriorating environmental conditions. Alterations in microbiota composition are consistent with the interpretation that they represent a consequence—or at least a correlate—of systemic stress, rather than its primary driver. Nevertheless, it is crucial to emphasize that this interpretation is based on observational and cross-sectional data; conclusive evidence regarding the directionality of this relationship will require prospective studies with longitudinal sampling.

The microbial community functions as an indicator, a “mirror” reflecting the physiological state of the host. However, the microbiota’s passive role is rapidly replaced by an active one.

### 3.2. Effects of the Altered Microbiota on the Intestine

Having changed under the influence of systemic factors, the microbiota transforms from a passive indicator into an active participant in pathogenesis. It begins to exacerbate the initial injury via several mechanisms, acting as a mediator.

The primary mechanism is the direct disruption of the intestinal barrier. The loss of commensals such as *Bifidobacterium*, *Lactobacillus*, and the probiotic strain *Escherichia coli* Nissle 1917 weakens epithelial tight junctions by reducing the expression of tight-junction proteins (ZO-1 and occludin) [1,17,18,19,24,44]. Simultaneously, overgrown pathobionts actively attack the barrier. *Clostridium perfringens* and pathogenic *E. coli* strains produce toxins that disrupt intercellular junctions [1,14,17,18,19]. *Helicobacter pylori* delivers the CagA protein into epithelial cells, further impairing junction integrity [45]. Even *Akkermansia muciniphila*, typically beneficial for maintaining mucosal thickness, begins to degrade the mucin layer under conditions of mucosal thinning, thereby aggravating the injury [1,18,21].

The second mechanism involves the amplification of inflammation and the induction of carcinogenesis. An increased proportion of *Proteobacteria* leads to elevated concentrations of lipopolysaccharides (LPSs) in the intestine, which act as potent proinflammatory stimuli [36,37]. Enterotoxigenic *Bacteroides fragilis* (ETBF) and *Fusobacterium nucleatum*, which proliferate under dysbiotic conditions, are directly associated with the development of colorectal cancer, chemoresistance, and recurrence risk [18,19,22,27,31,43,44,46].

The process by which microbial components, primarily LPS, penetrate through the compromised intestinal barrier into the internal environment is known as bacterial translocation [47,48,49]. This phenomenon is a central element of a broader pathological state referred to as enteral insufficiency syndrome or enteral distress syndrome [47,50,51]. It develops in response to severe systemic stress and is characterized by barrier dysfunction, overgrowth of pathobionts, and their translocation into the internal milieu, effectively turning the intestine into the “engine” of multiple organ failure [50,51]. This syndrome is a key mechanism that transforms a local disturbance into a systemic pathology, inducing systemic inflammatory response syndrome (SIRS), abdominal sepsis, and multiple organ failure [15,51,52]. In addition, bacterial translocation can contribute to surgical site infections (SSIs) [34,53].

**Figure 1:**
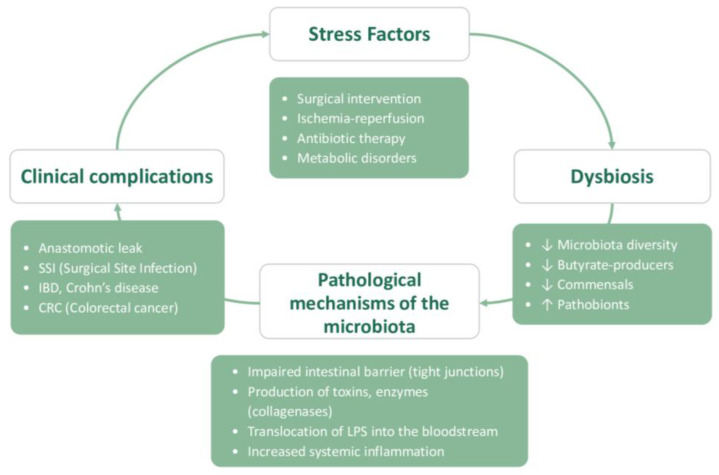
The role of the gut microbiota as a mediator in the vicious circle of postoperative complications. Surgical stress (1) induces dysbiosis (2), leading to intestinal barrier disruption (3). Subsequent bacterial translocation enhances systemic stress (4), perpetuating the cycle.

These processes create a self-reinforcing vicious circle, as illustrated in Figure 1: systemic stress → dysbiosis → barrier disruption → enhanced systemic stress. This mechanism likely underlies the pathogenesis of postoperative complications. For example, anastomotic leakage has been associated with increased abundance of *Acinetobacter lwoffii*, *Barnesiella intestihominis*, and *Hafnia alvei* [1,19,20,27]. The pathophysiology of anastomotic failure also involves *Enterococcus faecalis* and *Fusobacterium nucleatum*, which promote collagen degradation through the activation of matrix metalloproteinases (MMP9) [18,19,27]. Surgical site infections and sepsis are frequently caused by *Bacteroides*, *Enterococcus faecalis*, *Pseudomonas aeruginosa*, and *Staphylococcus* species [18,19,36,39]. Notably, *Clostridium difficile* proliferation is accelerated by excessive antibiotic use, leading to the suppression of other bacterial populations and the development of pseudomembranous colitis accompanied by diarrhea [18,41,44,53].

Thus, under adverse conditions, a shift in the dominant microbial populations occurs: commensal bacteria that normally maintain the intestinal barrier and produce anti-inflammatory metabolites (such as butyrate) are replaced by species capable of disrupting the intestinal wall, promoting inflammation, and predisposing to infectious and oncologic complications. This paradigm aligns with the concept that the microbiota not only signals underlying pathology but may also actively exacerbate it, potentially serving as a potent mediator that amplifies the inciting systemic stress. Nevertheless, the extent to which these microbial alterations drive pathogenesis—rather than merely reflecting it—remains challenging to quantify based on currently available data.

### 3.3. Controversial Roles of Microbial Species

Despite the growing body of evidence, the roles of many microorganisms remain ambiguous, and research findings often contradict one another. This highlights the complexity of microbial interactions and the dependence of microbial effects on context, strain-specific characteristics, and the host’s physiological state.

*Akkermansia muciniphila* provides a striking example of dual behavior. Its effects, protective or damaging, depend on the integrity of the mucosal barrier and the surrounding microbial environment [18,21]. Similarly, segmented filamentous bacteria (SFB) may exert both beneficial and detrimental influences: under normal conditions, SFB induce a protective Th17-mediated immune response, yet excessive stimulation can provoke autoinflammatory reactions [37,54]. Conflicting data also concern the families *Lachnospiraceae* and *Ruminococcaceae*: a marked decrease in their abundance is associated with severe dysbiosis and intestinal barrier dysfunction, whereas other studies report an increase in their numbers linked to postoperative complications [1,15,20,24,25].

Further inconsistencies arise from strain-level differences. For example, the role of the genus *Bacteroides* varies dramatically depending on whether the strain is enterotoxigenic or not: non-toxigenic strains can exert anti-inflammatory effects, whereas enterotoxigenic *Bacteroides fragilis* (ETBF) promotes carcinogenesis [18,19,22,30,55]. Data regarding the genus *Blautia* are also contradictory: its abundance decreases in Crohn’s disease and colorectal cancer, yet it increases in irritable bowel syndrome (IBS) and ulcerative colitis [21,40,56]. A similar inconsistency is observed for *Prevotella* species: while most studies report a decline after systemic stress, some have found the opposite [20,21,24,25,31,32]. Studies on *Fusobacterium* have also yielded conflicting results, with reports of both elimination following therapy and increased abundance [19,29,30,31]. The same holds for *Parabacteroides*: some publications describe a decrease, while others note an increase in abundance after surgery [19,55,57]. Divergent results have also been found for *Escherichia coli* and *Escherichia–Shigella*, showing both increases and decreases depending on the clinical context and specific strain [12,14,19,23,29,32,45].

The most paradoxical findings concern *Roseburia faecis*. On the one hand, it is enriched in patients without complications, suggesting its potential as a marker of favorable outcomes; on the other hand, it is abundantly represented in patients with surgery-related complications. This discrepancy may reflect strain-specific characteristics or environmental dependencies [14]. Even seemingly well-studied species show conflicting behavior. For instance, bacteria of the genus *Lactobacillus* are generally considered key commensals that strengthen the epithelial barrier. However, certain strains have been shown in vitro to increase epithelial permeability and even induce bacteremia [16,19].

Differences in the host’s clinical condition may also contribute to conflicting observations and outcomes. For example, the anti-inflammatory *Faecalibacterium prausnitzii,* whose abundance decreases during acute inflammation in inflammatory bowel disease (IBD) and in postoperative patients with complications, paradoxically increases in irritable bowel syndrome (IBS) [13,21]. Hence, its role may vary depending on the underlying pathology, whether it involves acute systemic inflammation or chronic low-grade dysfunction.

The effect of a given microorganism, thus, appears to depend on its strain, the surrounding microbial community, and the immune and metabolic status of the host. These findings strongly suggest that microbial function is not an intrinsic or immutable property of a species. Rather, it is context-dependent, determined by the host’s physiological state and the structure of the microbial ecosystem.

Key examples of context-dependent microbial behavior are summarized in Table 2 to illustrate how strain variability, disease type, and host condition can explain these paradoxes. Resolving these inconsistencies requires further research focused on strain-level microbiome analysis and assessment of the functional potential of microbial communities under various stress conditions.

We emphasize that taxonomic identification at the species or genus level fails to capture functional heterogeneity at the strain level; indeed, opposing clinical effects can manifest within a single species, dictated by strain-specific virulence and metabolic profiles. Furthermore, the relative abundance of microorganisms quantified via sequencing reflects community composition rather than metabolic activity, virulence factor expression, or causal contribution to disease pathogenesis; thus, inferring biological effects necessitates functional and metabolomic data.

## 4. Discussion

A substantial proportion of research investigating the role of the microbiota in surgical contexts has focused predominantly on cataloging taxonomic shifts—namely, the expansion of pathobionts and the depletion of commensals. However, this approach provides only a fragmented perspective, as it examines the microbiota in isolation from the host’s physiological state. Attempting to elucidate the nature of postoperative complications by merely enumerating “good” and “bad” bacteria is akin to analyzing a forest fire by counting the surviving and charred trees. This reductionist perspective risks overlooking the fire itself—the systemic stress induced by surgical intervention that destabilizes the entire intestinal ecosystem. Both enterocytes and resident microorganisms may be victims of a shared catastrophe; thus, casting either party as the sole aggressor represents a fundamental oversimplification. We propose conceptualizing host tissue injury and microbial shifts as interconnected components of a complex system, wherein the precise directionality of causality remains an open question requiring further investigation.

Data indicating that the preoperative microbiota composition correlates with the risk of anastomotic leakage and other postoperative complications do not contradict our conceptual framework—provided they are interpreted appropriately. The critical question is not whether this correlation exists, but rather what it precisely signifies. One plausible interpretation is that preoperative microbial shifts reflect pre-existing, and often subclinical, impairments in the intestinal wall—consequences of the underlying disease, prior therapies, or concomitant factors. Under this interpretation, the microbiota acts not as an independent pathogenic agent, but rather as a sensitive biomarker of an already compromised intestinal milieu that subsequently, under surgical stress, manifests as a clinical complication. Nevertheless, it must be acknowledged that this interpretation remains inferential. The available data do not preclude alternative explanations—namely, the possibility that specific microbial taxa actively contribute to primary intestinal barrier impairment even prior to surgery, or that bidirectional feedback mechanisms are operative from the outset. Definitive resolution of this causal question will require prospective studies incorporating serial assessments of both barrier function and microbiota composition before and after surgical intervention.

It is crucial, however, to distinguish between microbial markers that passively reflect microenvironmental injury prior to surgery and those that actively drive disease progression. The majority of preoperatively identified taxa serve merely as indicators of tissue hypoxia, whereas select species become active participants in pathogenesis once the barrier is breached—specifically by producing enzymes that degrade the collagen framework of the anastomotic suture line. A striking example is *Enterococcus faecalis*: experimental models have demonstrated that this pathobiont can directly cleave tissue collagen at the anastomotic site and activate host matrix metalloproteinase-9 (MMP-9) [58], while clinical data confirm a high rate of colonization of the anastomotic suture line by these specific strains in patients with anastomotic leakage following colorectal surgery [59]. It is the primary injury to the host barrier that “permits” the pathogenic actions of specific strains [58,59].

**Figure 2:**
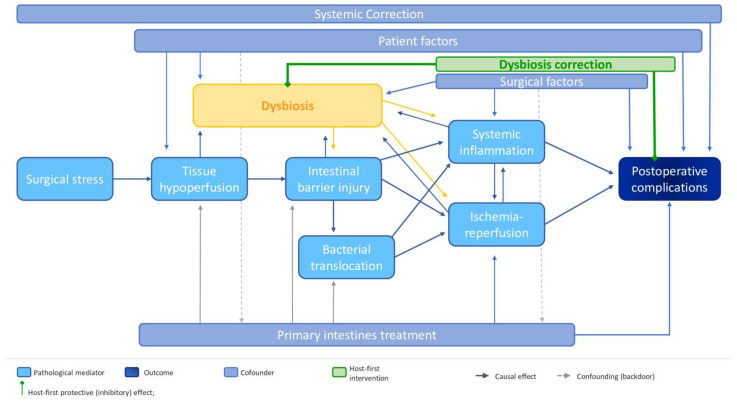
Hypothetical causal model of postoperative intestinal barrier failure (directed acyclic graph).

Validating this interpretation requires prospective studies that correlate preoperative assessments of intestinal wall histology and microcirculation with microbiota composition and subsequent clinical outcomes. We hypothesize that after statistical adjustment for the functional parameters of the intestinal wall, the direct association between preoperative microbial signatures and postoperative complications will be substantially attenuated. This does not diminish the utility of the microbiota as a predictor; rather, it can serve as a convenient, noninvasive indicator of the severity of pathophysiological alterations within the gut. However, therapeutic interventions should target not the microbial shifts themselves, but rather their underlying cause: impairment of the intestinal wall and its microcirculation. The proposed causal hierarchy is schematically illustrated in Figure 2.

It must be acknowledged that surgical factors—operative technique, tissue perfusion, anastomotic tension, blood loss, and operative time—can substantially confound the presumed associations between the microbiota and postoperative complications. The surgical procedure is a critical event driving intraluminal and intramural alterations: all these factors predominantly impact the pathophysiology of the intestinal wall itself—either directly through mechanical trauma or indirectly through the disruption of systemic hemodynamics and transcapillary exchange. This observation aligns with the hypothesis that luminal microorganisms largely adapt to the extreme conditions encountered intraoperatively, rather than initiating the pathophysiological cascade. However, this should not be construed as a definitive statement on causality. In the absence of prospective studies incorporating frequent longitudinal sampling and appropriate adjustment for surgical covariates, the relative contributions of host tissue injury and microbial factors remain undetermined. The “host-first” hierarchy we propose is advanced as a conceptually grounded and testable hypothesis, rather than an established biological fact.

Recognizing surgical factors as the primary determinants also necessitates a critical reappraisal of the direction of causality within the host–microbiota axis. Most studies implicitly cast microbial shifts as the initiators of pathology, categorizing specific taxa as inherently protective or pathogenic. However, the term “opportunistic pathogen” is frequently misunderstood: the operative word is “opportunistic,” not “pathogen.” It is the local host microenvironment that dictates whether a microbe remains a commensal or becomes deleterious. In the absence of tissue hypoxia, ischemia–reperfusion injury, or pharmacological destabilization, even classic pathobionts coexist peacefully with the host. What is often interpreted as microbial aggression is, in most cases, merely a secondary bacterial adaptation to a deteriorating habitat. Erosions and ulcers develop primarily as a consequence of ischemia and impaired oxygen delivery to enterocytes; thus, the subsequent microbial colonization of the damaged area is a consequence, rather than the cause, of the primary tissue defect. Similarly, intestinal paresis, which is often attributed to anaerobic toxins, is in fact a secondary event: bacterial overgrowth and toxin production become feasible only after motility has already been compromised by surgical trauma, anesthesia, or electrolyte imbalances. Thus, systemic stress first degrades the intestinal milieu, and the subsequent microbial response amplifies, rather than initiates, the pathological cascade. Until this temporal sequence is fully acknowledged, any therapeutic strategy targeting the microbiota without addressing host tissue resilience will remain unpredictable and, in the setting of acute surgical stress, likely futile.

This reconceptualization, if validated, will fundamentally alter the therapeutic paradigm. If the primary insult is indeed directed at the ecological niche itself—which remains to be conclusively demonstrated—therapeutic efforts should focus not on manipulating individual taxa, but rather on fostering conditions conducive to the natural restoration of the entire ecosystem. Conversely, if future studies demonstrate that microbial shifts in specific contexts can precede or independently precipitate intestinal wall injury, a more nuanced, context-dependent approach will be required. It has long been established that excessive antibiotic therapy causes even greater harm by further destroying the residual structure of the ecosystem and hindering its spontaneous restoration [36]. The most common and severe complication of prolonged antibiotic use is Clostridium difficile infection, which can lead to severe diarrhea, colitis, toxic megacolon, and death [58]. Moreover, uncontrolled antibiotic administration promotes microbial resistance [60]. The microbiome becomes a reservoir of resistance genes, greatly complicating the treatment of any subsequent bacterial infection [61,62]. Antibiotics also exert direct effects on host physiology. According to several studies, their use alters gene expression, protein activity, and more than 87% of all detected metabolites, thereby disrupting most metabolic pathways [63].

Beyond antibiotics, other classes of drugs routinely used in systemic therapy for comorbid conditions also impact the intestinal barrier. Life-saving interventions such as vasopressor (adrenergic) therapy for hemodynamic stabilization often come at the cost of pronounced splanchnic vasoconstriction and hypoperfusion, exacerbating ischemia–reperfusion injury of the mucosa [64]. General anesthesia produces similar outcomes: anesthetics induce vasodilation and hypotension, reducing intestinal perfusion, while compensatory fluid resuscitation increases the risk of intestinal wall edema [65]. Studies demonstrate that excessive infusion of saline solutions induces interstitial edema of the gut wall, compromising its barrier function [65,66,67]. Simultaneously, glucocorticosteroids administered to control systemic inflammation suppress local immunity and delay epithelial regeneration, thereby predisposing patients to postoperative infections [68,69]. Thus, each of these interventions, though aimed at preserving vital organ function, inflicts collateral damage upon the intestine, creating a chemically hostile and ischemic environment. The resulting pharmacological burden depletes the ecosystem’s capacity for self-repair and shifts the balance toward irreversible dysbiosis, rendering subsequent microbial correction attempts largely futile.

Prebiotic, probiotic, and synbiotic therapies are actively promoted in perioperative care, and several meta-analyses have reported reductions in the incidence of infectious complications, postoperative ileus, and hospital length of stay in specific cohorts of patients undergoing gastrointestinal surgery. These findings merit recognition: probiotics and synbiotics can indeed be beneficial in the preoperative setting or in patients with a relatively intact intestinal wall, where a favorable microbial environment can be established prior to surgical intervention—and in our own clinical practice, we share this positive perspective in appropriate scenarios. However, a detailed analysis of these meta-analyses reveals that the maximum benefit is observed in studies where probiotics were administered preoperatively or within the first few hours postoperatively—that is, before the onset of significant intestinal wall injury. In several studies, no significant differences in outcomes were found between the probiotic and control groups, while others reported an increased incidence of complications in the probiotic arm [70,71]. Current evidence generally demonstrates substantial inconsistency regarding the efficacy of probiotics in the setting of acute surgical stress [63]. The heterogeneity of protocols—involving different strains, dosages, initiation times, and types of surgery—makes it difficult to draw universal conclusions, as treatment effects are significant in some subgroups but absent or even detrimental in others. It is precisely this heterogeneity of effects that our analysis aims to elucidate. Under conditions of acute surgical stress—characterized by postoperative ileus, mucosal ischemia, edema, and impaired microcirculation—the intestinal wall no longer constitutes a stable habitat for exogenous microorganisms. Introducing exogenous bacteria into an inflamed, ischemic environment with an already compromised barrier may not only be ineffective but also paradoxically harmful. We propose reconsidering probiotics and synbiotics not as universal tools for dysbiosis correction, but rather as agents that support existing intestinal wall health—most appropriately utilized in the preoperative period or, in the postoperative phase, only after the restoration of barrier function. Validating this position requires stratified studies in which the condition of the intestinal wall—assessed, for example, by I-FABP or zonulin levels—serves as both an inclusion criterion and a stratification variable.

The context-dependence of microbial effects, exemplified by the “paradoxes” identified in our analysis (e.g., the dual role of *Akkermansia muciniphila* and *Roseburia faecis*), provides a compelling rationale for caution when interpreting associations between specific taxa and clinical outcomes. If a single microorganism can exert both beneficial and detrimental effects depending on the host’s physiological state—a phenomenon supported by experimental data but requiring further validation in human surgical populations—then the outcomes of interventions, whether antibiotics or probiotics, may prove inherently unpredictable. An antibiotic may eradicate a pathobiont while simultaneously creating an ecological niche for a more virulent organism; conversely, a probiotic strain that is beneficial in a healthy gut may behave differently in an inflamed postoperative environment. While this unpredictability does not preclude the possibility that carefully selected and timely administered probiotics may be beneficial in specific patient subgroups, it underscores the necessity for rigorous stratification and prospective testing.

We explicitly acknowledge that bidirectional interactions between the host and the microbiota remain a conceptual possibility that cannot be dismissed on the basis of currently available data. It is highly plausible that in certain clinical scenarios—particularly in the context of chronic inflammatory diseases or persistent dysbiosis—microbial products or metabolites may exert a direct deleterious effect on the intestinal epithelium, thereby initiating or perpetuating barrier dysfunction independent of systemic hypoperfusion or ischemia.

Nevertheless, in the context of acute surgical stress, a fundamental pathophysiological question arises: how could the luminal microbiota undergo pathological shifts—let alone exert a direct deleterious effect on the intestinal wall—without preceding alterations in the mucosal microenvironment? In order for bacterial enzymes to reach the collagenous scaffold of the anastomosis, toxins to disrupt intercellular contacts, and lipopolysaccharides to trigger a systemic inflammatory response, the structural integrity of the epithelial barrier must first be compromised. The intact intestinal epithelium, characterized by tight junctions, a mucus layer, and continuous enterocyte turnover, precludes the unrestricted translocation of luminal contents into the subepithelial space. While not discounting the possibility of reciprocal interactions in chronic or subacute settings, we posit that in the context of acute surgical stress, disruption of the intestinal habitat—whether due to ischemia, edema, pharmacological injury, or mechanical trauma—is a prerequisite for subsequent pathogenic microbial activity. This does not diminish the role of the microbiota as a mediator; rather, it assigns it its proper pathogenetic role—not as the primary aggressor, but as a potent amplifier of injury initiated by host factors. We fully acknowledge that this model requires direct experimental validation; accordingly, this review formulates a testable hypothesis, inviting the scientific community to subject it to critical scrutiny.

While acknowledging the importance of surgical factors as potential confounders, it is essential to clearly delineate their role within the proposed conceptual model. The impact of these variables on the incidence of postoperative complications is well documented: prolonged operative time is associated with a dose-dependent increase in complication rates [72], and excessive anastomotic tension is recognized as a key technical determinant that compromises perfusion and anastomotic healing [73]. Intraoperative contamination increases the risk of surgical site infections in direct proportion to the wound contamination class [74], whereas greater surgical volume and surgeon experience, conversely, are associated with reduced perioperative mortality [75]; furthermore, the implementation of Enhanced Recovery After Surgery (ERAS) protocols reduces complication rates and shortens the length of stay [76]. However, the critical question is not whether these factors exert an effect, but rather how precisely they mediate the association between the microbiota and clinical outcomes.

Upon closer examination, it becomes evident that all the aforementioned factors converge on a single final common pathway—the physiological state of the intestinal wall and its microcirculation. Prolonged operative time increases the duration of tissue ischemia, exacerbates the systemic inflammatory response, and promotes intestinal wall edema, directly impairing barrier function. Excessive anastomotic tension induces local ischemia at the suture line and facilitates the penetration of luminal microorganisms into the subepithelial space—but only if local perfusion is already compromised. Intraoperative contamination becomes clinically significant primarily when the protective mechanisms of the intestinal wall are impaired; otherwise, host immune and barrier defenses effectively prevent infection. Surgeon experience mediates its effects through all the aforementioned mechanisms: a more experienced surgeon typically achieves superior hemostasis, minimizes tissue trauma, and reduces operative time, thereby decreasing the extent of intestinal wall injury. Postoperative management protocols—including the timing of enteral nutrition initiation, fluid management strategies, and vasopressor regimens—also operate through a unified final mechanism: preserving or impairing adequate intestinal perfusion.

Thus, the proposed causal hierarchy does not negate the importance of surgical factors; rather, it integrates their impact into a unified pathophysiological framework. In this context, microbial shifts serve not as an independent cause of complications, but rather as a sensitive indicator of the extent to which cumulative intraoperative and postoperative insults have disrupted intestinal wall homeostasis. Nevertheless, we must candidly acknowledge that comprehensively capturing all these variables in clinical research presents a formidable methodological challenge: surgical technique is inherently difficult to quantify, and surgeon experience is challenging to objectify within multicenter protocols. For this reason, we strongly advocate incorporating detailed parameters of surgical technique and perioperative hemodynamics into all future predictive models—including the proposed GRI—during their validation phases.

In addition to intraoperative variables, postoperative outcomes are substantially influenced by the patient’s baseline status. The evidence base in this regard is robust: advanced age and frailty independently increase the risk of complications and mortality [77,78]; diabetes and obesity elevate the incidence of infectious and wound complications, respectively [79,80]; and preoperative nutritional risk, as assessed by the NRS 2002, predicts adverse outcomes following abdominal surgery [81]. Smoking and systemic immunosuppression—notably corticosteroid therapy—also independently increase the risk of infection [69,82]. Finally, the baseline microbiota composition itself holds predictive value: the characteristics of the mucosal microbiota at the anastomotic site correlate with the risk of anastomotic leakage [83]. While these associations are well established, what remains less clear is precisely how these baseline factors integrate into our proposed pathophysiological model.

Upon closer examination, all these factors converge on two interconnected final common pathways. The first pertains to the pre-existing state of the intestinal wall: villous and crypt integrity, mucus layer thickness, and the density of intercellular junctions. The second involves host reactivity: the capacity to mobilize defense mechanisms in response to surgical stress. It is precisely this reactivity that dictates the severity of intestinal barrier disruption across different patients exposed to an identical surgical insult.

The specific mechanisms are as follows. Advanced age and frailty are associated with attenuation of the epithelial layer, decreased crypt depth, reduced stem cell proliferative activity, a diminished proportion of butyrate-producing bacteria, and impaired immune reactivity. Diabetes mellitus exacerbates ischemic injury through diabetic microangiopathy, endothelial dysfunction, and hyperglycemia-induced increases in epithelial permeability. Obesity sustains chronic low-grade inflammation characterized by elevated production of TNF-α and IL-6, thereby reducing intestinal wall resilience. Nutritional insufficiency—particularly glutamine deficiency—directly compromises enterocyte energy metabolism, impairs tight junction protein synthesis, and delays epithelial regeneration. Smoking induces chronic vasoconstriction, impairs microcirculation, and amplifies oxidative stress. Immunosuppression diminishes the host’s ability to contain pathogens and eliminate translocated bacteria, effectively lowering the threshold for the transition from localized injury to systemic pathology. The baseline microbiota composition itself is a derivative of all the aforementioned conditions, reflecting the already established vulnerability of the ecosystem.

It is crucial to emphasize that all these factors exert their effects indirectly—by inducing baseline alterations in the intestinal wall and impairing host reactivity. They are not independent causal agents, but rather constitute the “backdrop” against which surgical stress unfolds. We must candidly acknowledge that comprehensively adjusting for all these variables in clinical research presents a virtually intractable challenge. For this reason, we strongly advocate interpreting all existing data with caution and designing future studies to incorporate stratification by these key patient-specific covariates.

In practical terms, this entails a shift in clinical focus: rather than attempting to determine whether *Akkermansia muciniphila* is a “friend” or “foe” in a specific patient, the clinician can assess mucus layer integrity, epithelial permeability, and microcirculatory status using readily available laboratory biomarkers. When encountering discordant microbiome data, we propose the following algorithm: assess barrier function by measuring serum zonulin and I-FABP levels. If these markers are within normal limits, the observed microbial shifts likely lack clinical significance. Conversely, if they are elevated, the therapeutic focus should shift toward barrier restoration—utilizing glutamine, butyrate, and microcirculatory optimization—rather than the eradication of specific taxa. Furthermore, serial I-FABP kinetics can serve as a surrogate endpoint for evaluating the efficacy of perioperative interventions: a failure of I-FABP levels to decline following probiotic administration should signal the need for strategy revision. In this context, the inherent discordance of microbiome data should not be viewed as an obstacle, but rather as a compelling rationale for transitioning to more robust and reproducible biomarkers of intestinal wall integrity.

Based on these insights, we advocate a fundamental shift in therapeutic focus from the luminal content to the intestinal wall itself. We propose a “host-first” strategy, where the primary goal is not to directly “correct” dysbiosis but to create conditions that favor endogenous recovery. This includes (1) barrier reinforcement using cytoprotective agents (e.g., zinc–carnosine [84] and rebamipide [85]); (2) epithelial nourishment with targeted metabolic substrates, such as glutamine and butyrate (as postbiotics) [86,87,88,89]; and (3) restoration of homeostasis via improved mucosal microcirculation [90].

Clinical evaluations of the efficacy of these nutritional substrates in surgical practice have yielded highly heterogeneous results. Meta-analyses of glutamine administration in abdominal surgery indicate a reduced incidence of infectious complications and shorter hospital stays following enteral or parenteral administration; however, large-scale studies in the intensive care unit (ICU) have revealed risks of deteriorating outcomes when excessive doses are administered to hemodynamically unstable patients [91,92,93]. The clinical scenario for butyrate is even more specific: its capacity to enhance anastomotic healing and increase the mechanical strength of the intestinal wall has been robustly demonstrated in meta-analyses of preclinical animal studies [94]; however, human clinical experience remains limited to the topical application of microencapsulated butyric acid for the relief of mucosal inflammation in diversion colitis and proctitis [95]. Therefore, within the context of the “host-first” strategy, these metabolic substrates are positioned not as definitively proven therapeutic agents, but rather as pathophysiologically grounded candidates for future targeted clinical trials.

It must be candidly acknowledged that, to date, no large-scale randomized controlled trials have directly compared the “host-first” strategy with microbiome-oriented interventions in surgical populations. This represents a significant “blind spot” in current knowledge. Nevertheless, routine practice in anesthesiology, critical care, and gastroenterology provides numerous examples of how barrier-oriented approaches are implemented indirectly with proven efficacy. Fluid therapy directed at optimizing microcirculation directly improves intestinal wall perfusion and mitigates hypoxic enterocyte injury. The titrated administration of vasopressors, carefully accounting for their impact on mesenteric blood flow, attenuates enterocyte death and prevents secondary dysbiosis. Enteral nutrition supplemented with glutamine, arginine, and omega-3 fatty acids has long been successfully utilized in critical care, although its direct comparison with probiotics in surgical settings remains unexplored. While none of these interventions are positioned as “microbiome-correcting,” they effectively create the conditions that allow the native microbiota to sustain itself. Our proposal does not entail inventing a fundamentally novel therapy, but rather systematizing and validating interventions that have already demonstrated indirect efficacy. The absence of direct comparative studies is not an argument against the “host-first” hypothesis; rather, it underscores the urgent need to conduct them.

Modern strain-level sequencing technologies and functional microbiomics have made remarkable strides; however, this does not alter our conclusion regarding the fundamental unpredictability of direct microbiome manipulation. This conclusion is not rooted in a disregard for technological advancements, but rather in the systemic properties of microbial communities—properties that cannot be bypassed even by the most precise sequencing. Current technologies can describe the microbial composition present in the gut at any given moment, but they cannot predict how these strains will interact with one another and with the host under the conditions of ischemia, inflammation, and the pharmacological burden characteristic of the acute postoperative period. The microbial community is a complex adaptive system whose behavior cannot be reduced to the sum of the properties of its individual strains. The bacterial mutation rate is approximately 10^−10^ per nucleotide per division; given a population size of roughly 10^14^ organisms in the human gut, the genetic diversity generated over even a single day is virtually infinite. Consequently, even a meticulously selected intervention—whether an antibiotic or a probiotic—can select for resistant or unexpectedly virulent variants within just a few days. Sequencing allows us to understand what went wrong, but it does not enable the reliable, real-time management of the microbial community in a critically ill patient. This is precisely why the therapeutic strategy must shift from attempting to “hack” the microbiome to establishing the conditions—adequate perfusion, barrier integrity, and normal motility—under which the patient’s own, evolutionarily adapted microbiota can recover autonomously.

The intestinal ecosystem possesses a remarkable capacity for endogenous resilience, an innate ability to self-stabilize and regenerate once the primary stressor is removed [36]. “Repairing the habitat” enables the host to naturally select and reestablish a symbiotic microbial community.

Nevertheless, the principles of a “host-first” approach and minimal intervention are not absolute. We postulate the existence of a “tipping point” in the process of ecosystem degradation—a threshold beyond which endogenous recovery may become impossible, and the intestine may transition into a state of self-destruction, functioning as an “engine” of systemic multiple organ failure. It must be emphasized that this threshold currently remains a purely theoretical construct, lacking direct experimental or clinical validation, a limitation we explicitly acknowledge. Equally plausible is the notion that in most clinical scenarios, no discrete threshold exists; rather, the relationship between intestinal injury and dysbiosis is continuous and bidirectional, characterized by gradual rather than catastrophic transitions. The existence and nature of such a threshold remain purely speculative and require direct empirical validation. Only when approaching this critical threshold might more aggressive interventions—such as targeted antibiotics or fecal microbiota transplantation—be justified: not to restore baseline homeostasis, but to prevent the catastrophic collapse of host physiology, particularly within the perimucosal microenvironment, including the crypts and villi.

Yet, the concept of a “tipping point” is not entirely unfounded; there are clinical scenarios in which an irreversible pathophysiological threshold is not merely a hypothesis, but an observable reality. In mesenteric thrombosis, there is a distinct temporal window (the “golden 6-h window”) during which revascularization can salvage the bowel. Once the thrombosis extends into the capillary bed and transmural necrosis develops, restoring blood flow no longer restores bowel viability. A similar sequence is observed in strangulating intestinal obstruction: mesenteric compression leads to venous stasis, followed by arterial ischemia and thrombosis of the intramural capillaries, after which relieving the strangulation fails to restore bowel viability. These examples demonstrate that the concept of an irreversible threshold has a tangible pathophysiological substrate: irreversible damage to the microcirculatory bed and the death of a critical mass of enterocytes. We extrapolate this logic to a broader spectrum of surgical conditions, hypothesizing that a similar threshold may exist for the intestinal ecosystem as a whole. However, for most clinical scenarios—such as elective resections, anastomotic procedures, and cardiac surgery—evidence for its existence is lacking, and its identification remains a task for future research. We must reiterate that the concept of a “tipping point” and its associated model of “rescue therapy” currently lack an empirical basis. The existence of a threshold beyond which endogenous recovery becomes impossible remains an unproven assumption requiring rigorous validation. Neither experimental data nor clinical observations currently substantiate the actual occurrence of such a transition in the majority of studied populations. We introduce these concepts as working hypotheses intended to stimulate scientific discourse and guide the design of future studies, rather than as clinical guidelines.

Regarding FMT as a tool for “rescue therapy,” robust evidence supports its efficacy in treating severe *Clostridioides difficile* infection (CDI) in the intensive care setting. In a series of 9 unstable critically ill patients with fulminant CDI, the primary cure rate following a single FMT session was 78% (7/9), enabling 88.9% of the cohort (8/9) to avoid emergency colectomy [96]. However, in the context of non-specific postoperative dysbiosis, FMT carries substantial safety risks: the administration of live bacteria across a compromised mucosal barrier can precipitate bacterial translocation and sepsis, as well as documented systemic inflammatory response syndrome. Notably, a case has been reported in which an entirely healthy, immunocompetent volunteer developed a severe SIRS-like syndrome—characterized by a fever of up to 39.1 °C, tachycardia, and leukocytosis (16.7 × 10^9^/L)—following the ingestion of encapsulated donor microbiota, necessitating emergency treatment with ciprofloxacin [97]. If such profound systemic reactions can occur in the setting of an intact intestinal barrier, the presence of a compromised bowel wall in surgical patients markedly amplifies the risk of systemic dissemination of infection. Consequently, within the framework of our proposed concept, the application of FMT is permissible only after the restoration of intestinal wall integrity [98]. Preclinical murine studies indicate that FMT combined with short-chain fatty acids can reduce mortality, restore microbial diversity, and attenuate markers of intestinal inflammation; however, extrapolating these findings to surgical patients warrants extreme caution and further investigation.

We propose a Gut Resilience Index (GRI) to operationalize this concept as a composite metric integrating dynamic parameters of both the microbiome and the host. Potential components may include (1) host barrier integrity markers (e.g., serum zonulin [99] ns intestinal fatty acid-binding protein (I-FABP [100])); (2) functional microbial ratios (e.g., butyrate producers to pathobionts and reductions in alpha diversity measured by Shannon or Simpson indices [1,101]); and (3) metabolic response signatures following surgery (e.g., the kynurenine-to-tryptophan ratio, balance of unsaturated vs. saturated lysophosphatidylcholines, hexadecanoylcarnitine levels [102], and fecal short-chain fatty acid profiles [103]). It is crucial to emphasize that at this stage, the GRI remains a purely theoretical construct. It lacks both biological and clinical validation; its predictive performance has not yet been evaluated, and clinical decision thresholds remain undefined. We introduce this index as a heuristic tool to structure future research, rather than as a ready-to-use diagnostic test.

Biomarker selection was guided by three criteria: pathophysiological rationale (the marker reflects the mechanisms of intestinal wall injury or repair), clinical feasibility (assessable in routine or easily implementable clinical practice), and dynamic sensitivity (demonstrating changes within hours to days in response to therapeutic interventions). For instance, I-FABP—a protein component of the index and a marker of enterocyte necrosis—is released into the systemic circulation within the first hours of ischemia, reliably correlating with the risk of subsequent anastomotic leakage [104], whereas a marked postoperative elevation in the kynurenine-to-tryptophan ratio, coupled with hexadecanoylcarnitine deficiency, enables the quantitative prediction of surgical complication severity [102]. To date, no completed studies have integrated all these parameters into a single composite score and validated it within a surgical population. We openly acknowledge this as a critical knowledge gap. Nevertheless, independent evidence supports the utility of each individual component. Furthermore, precedents of successful multi-component index development in related fields—such as the APACHE and SOFA scores, as well as the Intestinal Failure Index—demonstrate the fundamental feasibility of this approach.

**Figure 3:**
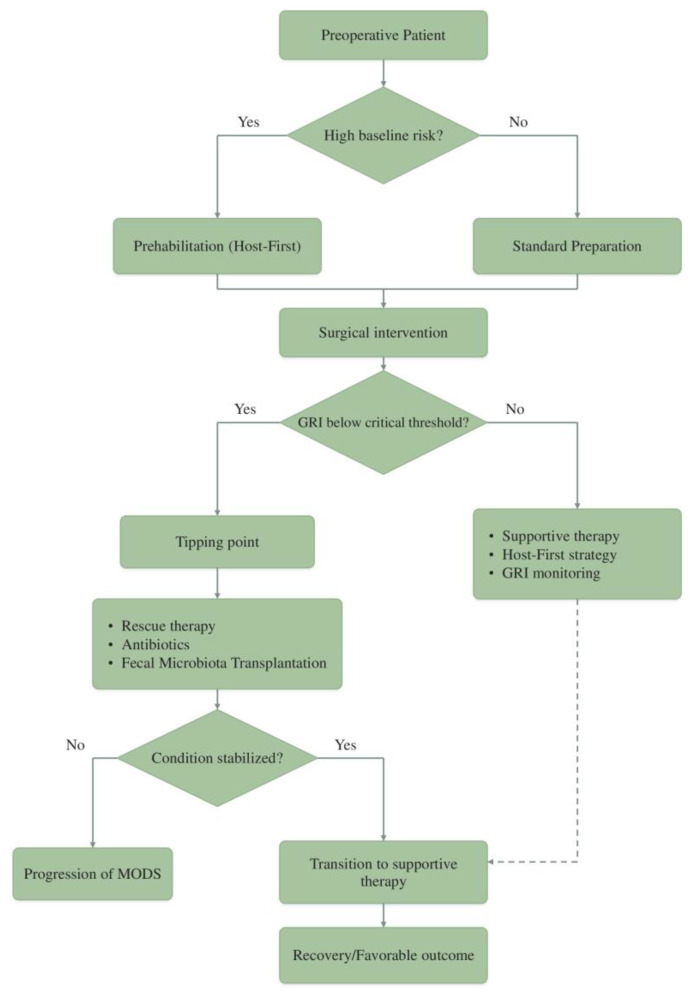
Proposed conceptual algorithm for patient management based on the assessment of intestinal resilience. This algorithm represents a hypothetical model designed exclusively to guide future research directions. It relies on the Gut Resilience Index (GRI) and the concept of the “tipping point” (critical threshold), neither of which has been validated to date; consequently, in its current form, it is not intended for clinical decision-making.

A sharp and sustained decline in the GRI below a validated threshold indicates proximity to the “tipping point”, signaling the need to transition from supportive management to active intervention. We developed a clinical decision-making algorithm integrating these concepts into a unified practical framework (Figure 3). It illustrates how patient stratification and therapeutic choice can be guided by the assessment of intestinal resilience, delineating two fundamentally distinct management trajectories depending on whether the hypothesized “tipping point” has been crossed, thus visualizing the transition from the supportive host-first strategy to an aggressive “rescue therapy.” We emphasize that, at this stage, the GRI remains purely a theoretical model. The index has not yet undergone clinical validation; the capacity of the proposed biomarkers to function collectively as a unified predictive model remains unproven; the relative weight of each component is undetermined; and clinically meaningful threshold values remain unknown. Consequently, this index is positioned as a conceptual framework for hypothesis generation rather than a tool ready for clinical application.

Any clinical application will require prospective validation through a stepwise pathway: (i) prospective observational cohort studies to establish the individual associations of candidate biomarkers with adverse postoperative outcomes; (ii) derivation of a weighted composite score using multivariable regression or machine learning; (iii) assessment of discriminative performance (area under the receiver operating characteristic [ROC] curve) and calibration using the Hosmer–Lemeshow test; (iv) determination of thresholds anchored to clinical outcomes; and (v) external validation in independent cohorts prior to the initiation of any interventional trials. Furthermore, future studies must incorporate safeguards against model overfitting—such as regularization or predictor reduction—as well as internal validation techniques (bootstrapping or cross-validation) and rigorous strategies for handling missing data, such as multiple imputation. Without these methodological safeguards, even the most promising predictive models frequently exhibit poor reproducibility when translated from research to clinical practice. We acknowledge that the final index, upon completion of all validation phases, may differ substantially from the initial conceptualization—potentially necessitating the replacement of specific biomarkers or the revision of their weights and thresholds. Nevertheless, we remain convinced of the heuristic value of the core concept—an integral assessment of gut resilience—as a tool capable of shifting the therapeutic paradigm in surgery.

Our model neither refutes nor replaces existing conceptual frameworks; rather, it integrates the missing link. The concept of intestinal failure traditionally focuses on the loss of digestive and absorptive functions, alongside impaired barrier integrity that leads to bacterial and endotoxin translocation. The concept of enteral distress syndrome emphasizes the role of membrane-destabilizing processes in enterocytes as a trigger for systemic endogenous intoxication [50,51]. The concept of bacterial translocation describes the mechanism by which microbial components traverse the intestinal wall into the systemic circulation [47,48,49]. All these concepts share a common limitation: they predominantly view the intestine as a passive target organ. Our model introduces three key additions. First, it shifts the focus from the intraluminal contents to the intestinal wall itself, positing that enterocytes, intercellular junctions, the capillary network, and lymphoid tissue are the primary targets of systemic stress. Second, it establishes a clear causal hierarchy: systemic stress → impaired microcirculation → enterocyte ischemia → destabilization of intercellular junctions → secondary microbiota alterations and translocation. While we do not deny the feedback effects of the microbiota, we maintain that without primary injury to the intestinal wall, pathological translocation across an intact barrier is impossible. Third, it introduces the principle of minimal intervention in the microbial community: microbiota maintenance should be achieved indirectly—by normalizing perfusion, preserving mucin layer integrity, and ensuring an adequate supply of metabolic substrates. In this context, the GRI is not merely another prognostic score, but a tool specifically designed to assess intramural status: enterocyte injury (I-FABP), barrier permeability (zonulin), the functional state of the microbiota, and the host’s metabolic response.

To translate the proposed algorithm into a practical clinical tool, its core hypotheses must undergo systematic validation. The first and most critical step is the validation of its diagnostic components, which requires prospective observational studies to develop and to standardize the Gut Resilience Index (GRI) and define the threshold of the “tipping point”. In parallel, the therapeutic efficacy of the algorithm’s branches must be confirmed using randomized controlled trials comparing the “host-first” approach with conventional treatment strategies. Finally, these efforts will be fundamentally based on a deeper understanding of the biological mechanisms of ecosystem breakdown and recovery at the strain level. Answers to these questions will pave the way for the development of novel diagnostic tools and therapeutic modalities targeting the intestinal barrier, the cornerstone of improved postoperative outcomes in surgical practice.

It is imperative to explicitly delineate the status of the proposed “host-first” strategy. To date, no randomized controlled trials have directly compared this strategy with microbiota-directed approaches in surgical populations. When we posit that the “host-first” strategy is pathophysiologically more sound, we are articulating a hypothesis grounded in a logical synthesis of available data, rather than stating an established fact. This assertion reflects our interpretation of the underlying pathogenetic mechanisms and is not substantiated by direct evidence of comparative efficacy. The primary objective of the present work is not to propose a ready-to-use clinical protocol, but rather to catalyze the research agenda and stimulate scientific discourse. We propose a conceptual model that we believe merits rigorous investigation, yet one that remains open to critique, refinement, and, if necessary, falsification. In the absence of direct comparative trials, any claims regarding the superiority of one strategy over the other would be premature. Thus, we position this work not as a definitive clinical guideline, but rather as an invitation to scholarly dialogue and a foundational starting point for future investigations.

We acknowledge that integrating data from such highly heterogeneous clinical and experimental models carries the risk of unwarranted generalization. The pathophysiology of inflammatory bowel disease, ischemia during cardiac surgery, mechanical obstruction in abdominal surgery, and experimental stress in animal models differ substantially in their etiology, temporal dynamics, and baseline risk. Moreover, interspecies differences in microbiota composition between animal models and humans further limit the direct extrapolation of preclinical data.

Nevertheless, beneath this apparent heterogeneity lies a shared underlying substrate. Regardless of the inciting cause, all these models converge on a single unifying feature: injury to the intestinal wall, compromise of its microcirculation, dysregulation of the immune system, and disruption of intramural innervation. Irrespective of its baseline diversity, the microbiota adheres to universal principles of ecological self-regulation: reduced diversity, depletion of butyrate-producing commensals, and expansion of pathobionts consistently occur whenever the local mucosal microenvironment becomes adverse. Thus, the heterogeneity of these models does not weaken, but rather strengthens, our central argument: independent of the underlying etiology, the physiological state of the intestinal wall serves as the critical pathophysiological nexus.

However, it is imperative to clarify that the proposed unified model may not be equally applicable across all clinical contexts. A patient with long-standing inflammatory bowel disease presents with a fundamentally altered intestinal wall and a microbiota adapted to this compromised milieu, whereas a patient undergoing cardiac surgery sustains acute ischemic injury superimposed on a previously healthy bowel. The differences in chronicity, severity, and reversibility of the intestinal injury necessitate a tailored approach. Consequently, rigorous validation of the proposed model requires homogeneous cohorts, strictly standardized for the type of surgical intervention, baseline intestinal status, and perioperative management protocol.

We acknowledge that the narrative structure of this review purposefully emphasizes the “host-first” hypothesis and may create the impression of confirmation bias. This reflects a deliberate choice to coherently articulate an alternative conceptual framework, rather than to disregard competing interpretations.

Several studies challenge or complicate the proposed model. First, multiple studies have demonstrated that specific strains of *Enterococcus faecalis* and *Fusobacterium nucleatum* can directly injure the intestinal epithelium and the collagenous scaffold of the anastomosis, even in the absence of overt ischemia [58,59]. Notably, the FadA adhesin of *F. nucleatum* binds to E-cadherin, thereby disrupting the integrity of epithelial intercellular junctions [105]. Second, evidence indicates that preoperative microbiota composition correlates with the risk of complications independently of traditional risk factors, raising the possibility of a primary pathogenic role for the microbiota [83]. For instance, the gut microbiome profile has been shown to influence the risk of anastomotic leakage and recurrence rates following colorectal surgery [106]. Third, experimental evidence demonstrates that inoculating intact animals with specific pathobionts can induce barrier dysfunction in the absence of prior ischemic injury; specifically, oral colonization of intact mice with enterotoxigenic *Bacteroides fragilis* elicits persistent colitis and intestinal barrier disruption [107], suggesting the potential for primary pathogenicity of the microbiota under specific conditions.

These observations merit careful consideration and are in no way dismissed. In our view, they do not invalidate the overarching premise: for most microbially mediated injurious effects to manifest, the barrier must first be compromised. Nevertheless, we acknowledge that the boundary between “primary” and “secondary” injury may be less distinct than our model posits—particularly in the context of chronic inflammatory conditions or in the presence of highly virulent strains. We firmly believe that the collision of competing interpretations represents the most productive path toward scientific truth, and we welcome the critical scrutiny of our proposed hypothesis.

In concluding this discussion, we deem it imperative to reiterate that all central concepts presented in this work—the GRI, the “point of no return,” and “rescue therapy”—remain entirely hypothetical. None has achieved biological validation, undergone clinical evaluation, demonstrated predictive performance, or yielded established threshold values. We propose these constructs not as ready-to-use tools, but rather as a conceptual framework for future research. Their primary value at this stage lies not in immediate clinical applicability, but in their capacity to generate novel questions and direct the scientific community’s efforts toward the empirical testing of new hypotheses.

### Limitations

This review has several limitations that must be considered when interpreting the findings. The narrative, as opposed to systematic, nature of the methodology inherently carries a risk of selection and interpretation bias. Although literature selection was performed independently by two authors, with discrepancies resolved via consensus, we cannot entirely exclude the influence of interpretive bias during data synthesis. While we endeavored to include studies representing both corroborating and alternative perspectives, a formal risk-of-bias assessment was not conducted, which remains a standard limitation of narrative reviews.

This review synthesizes data from studies of fundamentally disparate designs, including animal models, in vitro experiments, human observational studies, randomized controlled trials, and meta-analyses. These sources vary considerably in their evidentiary strength. Mechanistic claims derived solely from animal or in vitro models should not be equated with clinically validated facts; many pathophysiological patterns observed under experimental conditions fail to translate to human clinical studies. Furthermore, while human observational studies can identify associations, they cannot establish causality. Where we cite preclinical data or observational associations to support mechanistic assertions, readers must account for this hierarchical context of evidence. Randomized controlled trials remain the only study design capable of establishing causal inferences, yet such trials are currently scarce in the domain of barrier-oriented interventions within surgical populations.

The inclusion of literature from heterogeneous clinical contexts—such as gastrointestinal surgery, cardiac surgery, inflammatory bowel disease, and critical care—limits the direct extrapolation of our findings to specific surgical populations. Pathophysiological mechanisms elucidated in one clinical model are not necessarily applicable to another. For instance, a patient undergoing elective colorectal resection, a patient in a cardiac surgery intensive care unit, and a patient experiencing an inflammatory bowel disease flare possess fundamentally distinct baseline states regarding intestinal barrier integrity, microbiota composition, immune status, and pharmacological burden. The degree to which the conceptual model proposed herein applies to each of these populations varies and, in some cases, remains unknown. We utilized data from these diverse contexts to construct a unified pathophysiological framework rather than to formulate population-specific clinical guidelines. Readers should therefore interpret generalizations beyond the specific clinical contexts detailed in the cited literature with caution, treating the applicability of the proposed model to distinct surgical specialties as a separate question requiring targeted investigation.

A substantial proportion of the included data is derived from animal studies and in vitro experimental models. While these data provide a mechanistic rationale, their translational value is inherently limited, as many pathophysiological patterns observed in animal models fail to be replicated in human clinical trials. Wherever we rely on preclinical data, we have made every effort to explicitly state so in the text. Furthermore, the narrative design of this review precludes the quantification of effect sizes and the performance of meta-analytic calculations; consequently, the conclusions presented reflect a qualitative synthesis of the available evidence.

Finally, several central tenets of this review—including the concept of reverse causality, the context-dependent effects of microorganisms, the paramount role of surgical factors, and the concept of the “tipping point”—are inherently hypothetical. These concepts are formulated on the basis of logical analysis and indirect evidence, rather than being substantiated by direct prospective studies. We openly acknowledge the speculative nature of these propositions and present them as working hypotheses that require empirical verification, rather than as established facts.

## 5. Conclusions

The traditional view of dysbiosis as a primary cause of postoperative complications requiring direct microbial correction is incomplete and, we contend, one-dimensional. Drawing on this analysis, we propose a paradigm shift that merits serious consideration: dysbiosis may be more productively conceptualized not as an independent disease entity, but as a manifestation—or at least a correlate—of a compromised habitat, specifically the intestinal wall. Within this conceptual framework, the key determinant of clinical outcomes may lie not in the taxonomic composition of the microbiota per se, but in the functional resilience of the epithelial barrier—its capacity for self-regulation and regeneration under surgical stress. Preoperative microbial shifts that correlate with postoperative complications may be plausibly interpreted not as evidence of a causal role for the microbiota, but as biomarkers of an already compromised intestinal wall. Surgical factors—operative technique, tissue perfusion, and blood loss—are likely the primary determinants of intramural changes, whereas the microbial response represents a secondary adaptation to a deteriorating habitat rather than the initiator of the pathophysiological cascade. Nevertheless, we emphasize that this proposed hierarchy remains a working hypothesis. The predominantly observational nature of available human data precludes definitive conclusions regarding causality, and we cannot exclude the possibility of bidirectional or simultaneous mechanisms operating in many clinical scenarios.

The “host-first” strategy, which focuses on protecting barrier function, restoring microcirculation, and providing nutritional support to enterocytes, appears more pathophysiologically sound than direct correction of the microbiota’s composition. While probiotics and synbiotics may be beneficial in the preoperative setting or when the intestinal wall is relatively intact, their application under conditions of acute surgical stress remains unpredictable and necessitates stratification based on barrier function status.

Nevertheless, it is crucial to emphasize that at this stage, the proposed model remains a promising hypothesis rather than an established fact. Many tenets of this review—reverse causality, the context-dependent effects of microorganisms, and the paramount role of surgical factors—are inherently hypothetical and require rigorous empirical validation.

The proposed Gut Resilience Index (GRI) and the concept of the “tipping point” should be regarded as theoretical constructs designed to structure future research, rather than as ready-to-use clinical tools. The GRI has not undergone clinical validation; threshold values remain undefined, and the predictive value of the proposed biomarkers as an integrated model is unproven. Future research should focus on the prospective validation of GRI components, the determination of the “tipping point” via ROC analysis and cross-validation, and the conduct of randomized controlled trials comparing the “host-first” strategy with the standard of care. The primary conclusion of this work is not to assert an absolute truth, but rather to formulate a testable hypothesis and propose specific pathways for its verification. We urge the scientific community to view the proposed concept not as a finalized clinical protocol, but as a working hypothesis that remains open to critique, refinement, and empirical testing.

## Figures and Tables

**Table 1:** Functional roles and clinical significance of key microbial groups in dysbiosis.

Functional Group	Representatives	Primary Role in Eubiosis	Changes Under Stress	Pathogenic Mechanism (Mediator Role)	Clinical Significance/Associated Conditions
I. Butyrate Producers and Barrier Protectors (Commensals)	*Faecalibacterium prausnitzii*, *Roseburia*, *Bifidobacterium*, *Lactobacillus*, *clusters Clostridium IV/XIVa*	Production of SCFAs, nourishment of colonocytes, strengthening of tight junctions, and immune regulation	↓↓ Sharp decrease	Weakening of metabolic defense and epithelial barrier integrity	IBD, increased risk of anastomotic leak, and postoperative complications
II. Inflammation Inducers and Pathobionts	*Enterococcus*, *Staphylococcus*, *Enterobacteriaceae (E. coli*, *Klebsiella)*, *Proteobacteria*	Present in low titers; controlled by commensals	↑↑ Significant increase	LPS production, translocation, biofilm formation, and induction of systemic inflammatory response syndrome	SSI, sepsis, anastomotic leak, and multiple-organ dysfunction syndrome
III. Specific Modulators of Carcinogenesis and Tissue Destruction	*Fusobacterium nucleatum*, *B. fragilis (ETBF)*, *P. aeruginosa*	Absent or in minimal quantities (for F. nucleatum).	↑ Emergence and/or growth	Pro-oncogenic effects, production of toxins (BFT), enzymes (collagenases and elastases), and tissue degradation	CRC, chemoresistance, and anastomotic leak
IV. Microbes with a Pronounced Context-Dependent Role	*Akkermansia muciniphila*, *Faecalibacterium prausnitzii*, SFB	Maintenance of the mucin layer, anti-inflammatory effects, and induction of Th17 response	↔ Contradictory changes	Degradation of the barrier when it is thin; provocation of autoimmunity; and paradoxical increase in IBS	Metabolic syndrome, autoimmune diseases, and IBS vs. IBD

**Table 2:** Examples of the context-dependent functions of the gut microbiota.

Microorganism/Phenomenon	Observation 1 (Expected or “Positive” Effect)	Observation 2 (Paradoxical or “Negative” Effect)	Likely Explanation for the Paradox (Context)
*Akkermansia muciniphila*	Maintains and restores the mucus barrier.	Degrades the mucus barrier.	Host status: The effect depends on the initial thickness of the mucin layer.
*Faecalibacterium prausnitzii*	A marker of health; decreases during acute inflammation (IBD and complications).	Increases in chronic dysfunction (IBS).	Type of pathology: The response depends on the nature of the disease (acute systemic inflammation vs. local dysregulation).
*Roseburia faecis*	Enriched in patients without postoperative complications.	Abundantly present in a group of patients with complications.	Strain-level differences: Different strains of the same species can have opposite properties.
*Enterococcus* spp.	Some strains are used in probiotics.	They are a leading cause of SSI, anastomotic leak, and sepsis.	Strain-level differences: Functions differ dramatically at the strain level (presence/absence of virulence factors).
*Lactobacillus* spp.	Key commensals that strengthen the gut barrier.	Some strains increase epithelial permeability in vitro; associated with growth in CRC tissue.	Strain- and species-level differences: Effects are extremely heterogeneous within the same genus.

## Data Availability

No new data were created or analyzed in this study. Data sharing does not apply to this article.
